# Deciphering comprehensive profiles of pathogenies and resistome of pork using integrating metagenomic and isolation strategies

**DOI:** 10.1002/imo2.70004

**Published:** 2025-02-25

**Authors:** Lianwei Ye, Qiao Hu, Tao Zang, Yaling Wang, Heng Heng, Edward Wai Chi Chan, Sheng Chen

**Affiliations:** ^1^ Department of Infectious Diseases and Public Health, Jockey Club College of Veterinary Medicine and Life Sciences City University of Hong Kong Kowloon Hong Kong China; ^2^ State Key Lab of Chemical Biology and Drug Discovery and the Department of Food Science and Nutrition The Hong Kong Polytechnic University, Hung Hom Kowloon Hong Kong China; ^3^ Shenzhen Key Lab for Biological Safety Control The Hong Kong Polytechnic University Shenzhen Research Institute Shenzhen China

## Abstract

The pork microbiome was investigated using an integrated approach combining isolation and metagenomic sequencing methods to comprehensively analyze the pathogens and resistome on pork surfaces. The study revealed a large number and diversity of pathogens and resistance genes, potentially originating from air, transportation, water, or cross‐contamination. These findings underscore the importance of implementing multifaceted food surveillance strategies to monitor and mitigate these risks effectively.

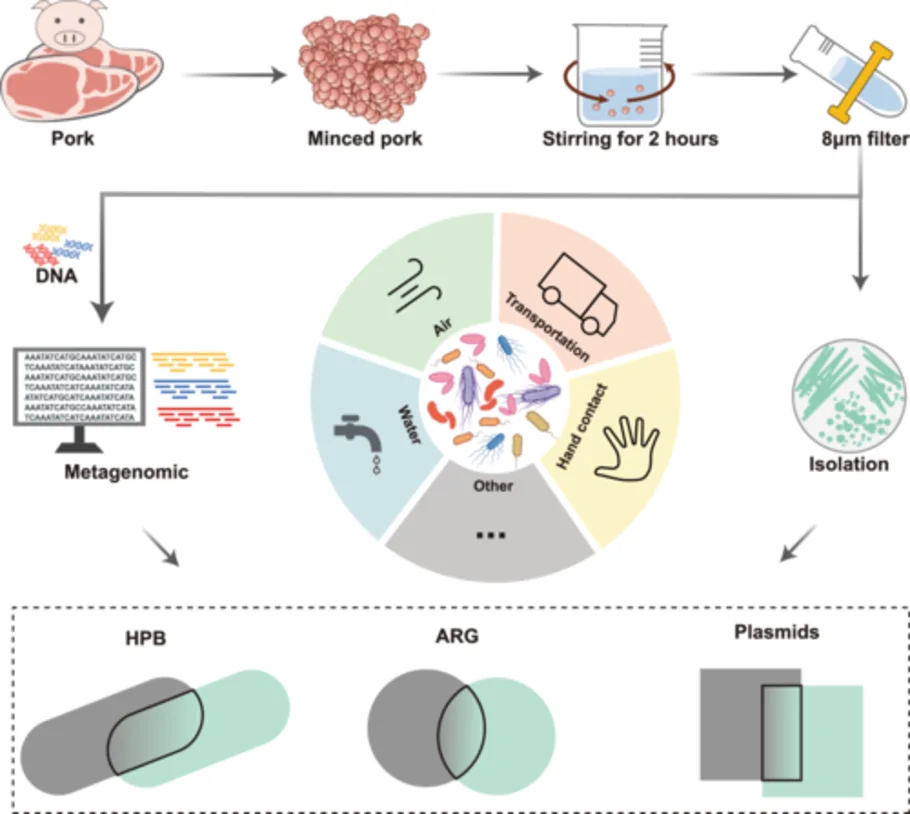

To the editor,

Pork is a significant source of foodborne pathogens, contributing to food poisoning. Meat products, including chicken, pork, and beef, are among the most common sources of pathogens, with global consumption projected to reach 376 million tons by 2030 [1, 2, 3]. Pork, compared to other meats, carries a higher risk of antimicrobial‐resistant bacteria, particularly *Klebsiella pneumoniae* and *Enterococcus spp*., which are resistant to critical antibiotics. While poultry is linked to *Campylobacter* and *Salmonella*, pork is associated with multidrug‐resistant *Enterococcus* and *Klebsiella*, increasing the risk of foodborne infections. This highlights pork's unique food safety risks compared to beef or poultry. Some pathogens carry antimicrobial resistance genes (ARGs), posing a multidrug resistance (MDR) threat. For instance, *Salmonella* strains isolated from pork in Jiangsu [4], China, exhibited high resistance to aminoglycosides (88.21%), tetracyclines (90.24%), and florfenicol (91.87%), harboring genes such as *strA*, *bla*_*TEM‐1*_, and *floR*. Traditional isolation methods have been instrumental in identifying human pathogenic bacteria (HPB) on pork, including *Escherichia coli*, *Salmonella enterica*, *Vibrio parahaemolyticus*, and *Staphylococcus aureus* [5, 6, 7, 8, 9]. These approaches have revealed ARGs like *bla*_*CMYs*_, *mcr‐1*, and novel plasmids, underscoring their role in uncovering new ARG variants [9, 10, 11, 12]. However, metagenomic sequencing offers a more comprehensive approach to studying microbial diversity. While 16S metagenomic studies, such as those by Koo et al. [13], identified abundant bacteria like *Pseudomonas* in ground pork, limited research exists on pork surface microbiomes using next‐generation sequencing. Challenges, such as extracting bacterial DNA from pork surfaces, hinder broader application. In our study, we integrated isolation and metagenomic methods to analyze the microbiome and resistome on the surface of pork from Hong Kong wet markets. Among 10 samples, Firmicutes and Proteobacteria were dominant, with 5 HPBs identified. Metagenomic analysis revealed 13 metagenome‐assembled genomes (MAGs), including pathogens like *Staphylococcus kloosii*. Isolation‐based methods identified 77 strains spanning 14 species, with *E. coli* and *Klebsiella* spp. predominating. Notably, some species like *Enterococcus spp*. and *S. enterica* were absent in metagenomic data but identified through isolation. Novel species, such as *Enterobacter kobei* and *Klebsiella africana*, were reported on pork surfaces for the first time. Metagenomic analysis uncovered 23 ARG types (621 subtypes), including clinically significant ones like *bla*_*NDMs*_ and *tet(X4)*. Isolation methods revealed 16 ARG types (134 subtypes), with 81 unique to this approach. These findings highlight the co‐occurrence of ARGs and virulence factor genes (VFGs) in HPBs, underscoring the complexity of antimicrobial resistance in foodborne pathogens.

## 1 RESULTS AND DISCUSSION

### Microbial communities of pork surface microbiome

**Figure 1:**
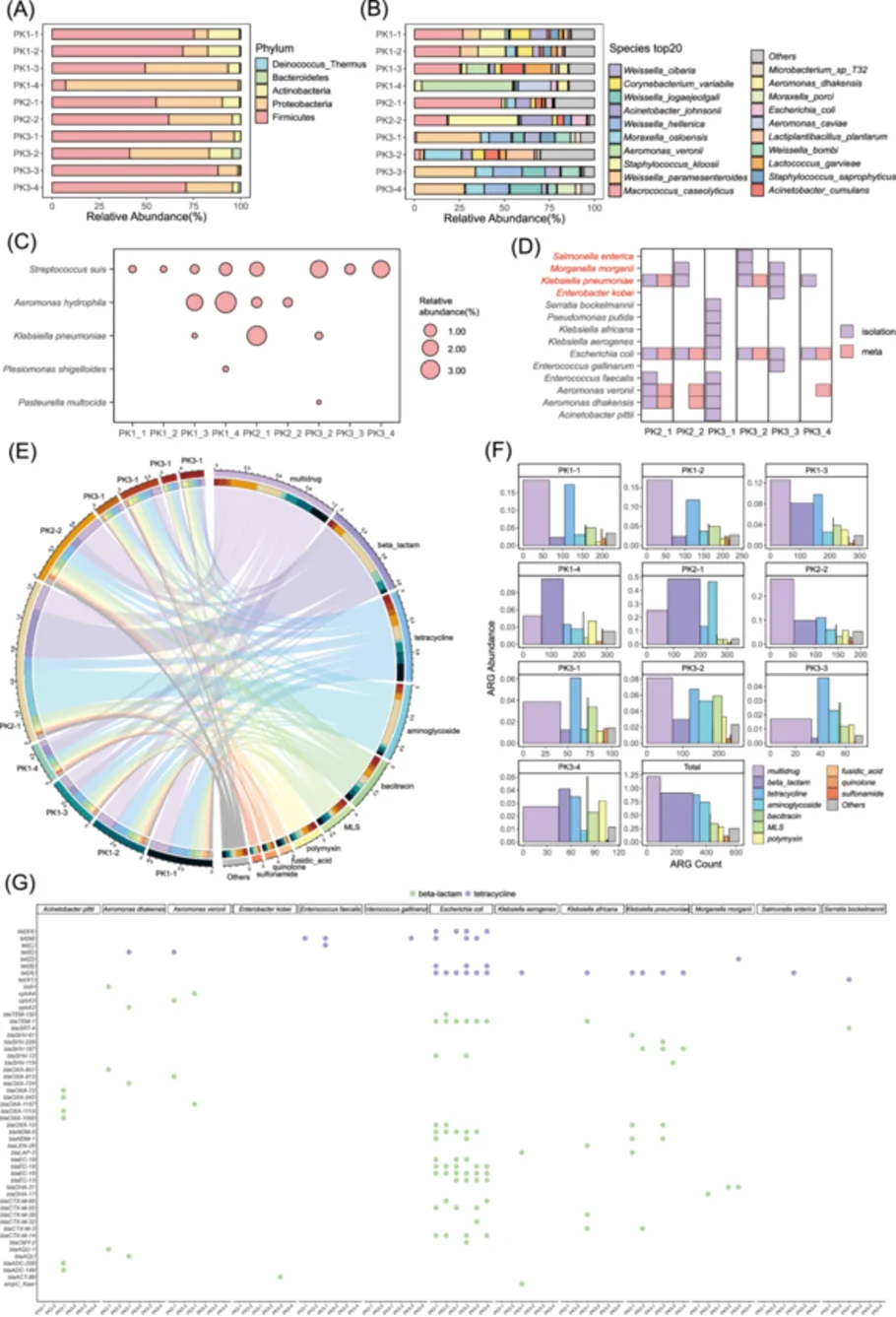
Comprehensive analysis of microbial diversity and antibiotic resistance in pork samples. (A) Phylum: A stacked bar chart representing the relative abundance of phylum found in different samples. (B) Species TOP20: A similar stacked bar chart as in (a), but detailing the top 20 species. (C) Human pathogenic bacteria (HPB) Species relative abundance (%): A bubble chart indicating the percentage of HPB species' relative abundance in various samples. (D) A heatmap showing the presence or absence of pathogens in different samples based on the isolation method (Purple indicates the species detected through isolation, while pink indicates the species identified via metagenomic analysis). HPB species are shown in red. (E) Antimicrobial resistance genes (ARGs) Relationships: A circular plot representing the relationships between different ARG types and sample locations, with lines connecting them indicating their associations. The outer ring of the circle is divided into segments labeled with different ARG types and sample locations. (F) ARG Abundance: A bar chart showing the abundance of ARGs and the number of types of ARGs subtypes in each sample. (G) Distribution of ARGs in Isolates: Colored markers within each column represent different ARG genes identified in bacterial strains from that sample. Strains include *Acinetobacter pittii*、*Aeromonas dhakensis*、*Aeromonas veronii*、*Enterobacter kobei*、*Enterococcus faecalis*、*Enterococcus gallinarum*、*Escherichia coli*、*Klebsiella aerogenes*、*Klebsiella africana*、*Klebsiella pneumoniae*、*Morganella morganii*、*Pseudomonas putida*、*Salmonella enterica* and *Shewanella bockelmannii*.

Metagenomic analysis identified the microbial communities on pork surfaces collected from wet markets in Hong Kong. Ten samples collected from MongKok Market, Ho Hing Sun Kee Meat Company, and New Generation Fruits & Vegetables were analyzed (Table S1), revealing Firmicutes and Proteobacteria as the dominant phyla, although their prevalence varied across the samples (Figure 1A). Firmicutes dominated most samples, except for PK1‐4 and PK3‐2, where Proteobacteria prevailed. Actinobacteria exceeded 10% abundance in PK1‐1, PK2‐1, and PK3‐2 (Table S2). At the genus level (Table S3), *Weissella*, *Staphylococcus*, and *Moraxella* were consistently dominant. Genera such as *Microbacterium*, *Acinetobacter*, *Aeromonas*, *Escherichia*, *Macrococcus*, and *Corynebacterium* were detected in more than eight samples. The number of genera per sample ranged from eight (PK1‐4) to 45 (PK3‐3). A high diversity of species is observed, with each sample carrying different species, which vary in their relative abundances (Figure 1B). HPB was detected in all samples, comprising five species (Figure 1C). Pathogens such as *K. pneumoniae*, *Enterococcus faecalis*, and others appeared sporadically.

Isolation methods identified 76 strains of four pathogenic bacteria across six samples. Dominant species included *Klebsiella spp*., with isolates such as *E. kobei*, *K. africana*, and *Salmonella enterica* (Figure 1D). Sample PK3‐1 hosted the highest diversity with nine strains, while PK2‐1 and PK3‐3 had five strains each. Isolated strains like *E. faecalis* and *E. gallinarum* were absent in metagenomic data, indicating that the isolation method could detect low‐abundance bacteria. While *E. coli* was detected in all samples by isolation, it was noted in metagenomic data only in some. Similarly, *K. pneumoniae* showed discrepancies between the two methods. Our combined analysis of pork surfaces revealed *K. pneumoniae* and other human pathogenic bacteria, which aligns with the findings of Emamjomeh et al. [14], who detected *Klebsiella* at the genus level. However, our species‐level identification uncovered greater diversity, revealing additional pathogens not detected by traditional methods. The discrepancy between isolation and metagenomic detection of strains like *Enterococcus spp*. and *K. aerogenes* could be due to their low abundance in metagenomic datasets. While metagenomics offers a broad overview, isolation detects low‐abundance or fastidious species [15]. Combining both methods provides a more comprehensive understanding of microbial diversity and food safety risks.

### Examination of abundance and diversity of ARGs

**Figure 2:**
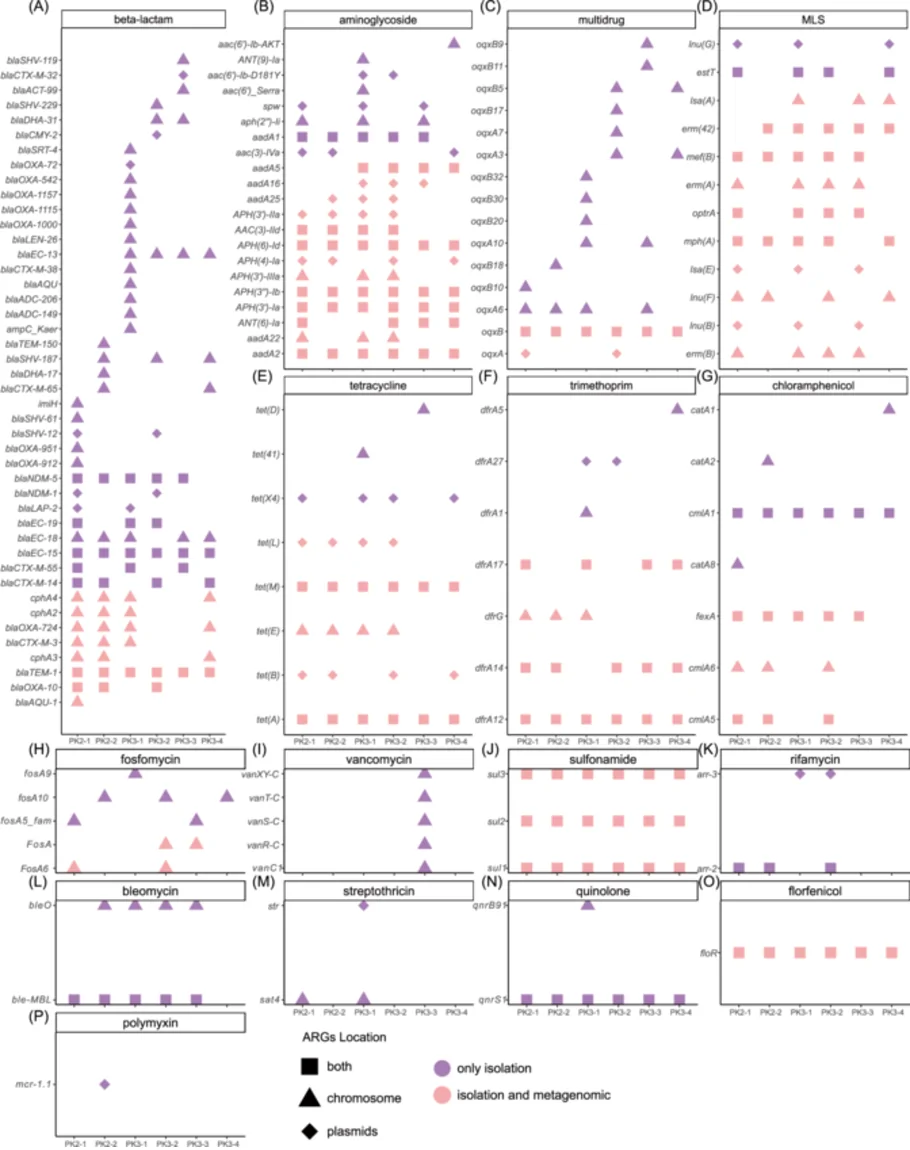
Distribution, location, and phylogenetic analysis of antimicrobial resistance genes (ARGs) in Isolation and MAGs. (A–P) ARG distribution by antibiotic class: This panel series illustrates the distribution and location of various ARGs associated with different classes of antibiotics. Each panel (A–P) represents a specific class of antibiotics. The *y*‐axis lists the ARGs, while colored symbols indicate the location and methods used to identify these genes. A diamond symbol represents ARGs located in plasmids. Color coding: purple indicates genes detected only from isolated strains, while red indicates genes detected in both isolated strains and metagenome‐assembled genomes (MAGs).

Metagenomic analysis of pork microbiomes revealed a high diversity and abundance of ARGs, identifying 23 primary ARG types and 621 subtypes (Table S4). Among these, multidrug, beta‐lactam, and tetracycline resistance genes were the most prevalent, with beta‐lactam resistance genes, particularly *bla*_*NDM*_, *bla*_*OXA*_, and *bla*_*CTX‐M*_ subtypes, dominating across samples (Figure 1E,F). Colistin resistance genes (*mcr‐1* to *mcr‐5*) were also detected, albeit at lower relative abundances. Diversity analysis showed significant variation in ARG profiles between samples, reflecting heterogeneity in microbial communities influenced by environmental factors and meat processing conditions. To complement the metagenomic findings, 76 bacterial isolates from pork samples underwent genomic characterization, revealing additional resistance determinants (Figure 1G). Notably, plasmid‐borne genes, including *bla*_*NDM‐5*_, *bla*_*CTX‐M‐15*_, *mcr‐1*, and *tet(X4)*, were detected, signifying their potential for horizontal gene transfer (Figure 2A–P). *Bla*_*NDM‐5*_, a carbapenemase gene, confers resistance to carbapenems, underscoring its significance in foodborne pathogens. *Tet(X4)*, associated with tigecycline resistance, raises concerns about its emergence in foodborne and clinical contexts.

Furthermore, the comparative analysis demonstrated distinct ARG compositions between metagenomic datasets and isolated genomes, highlighting the value of combining both approaches for comprehensive ARG profiling. These findings underscore the pork microbiome as a reservoir of ARGs, some of which are clinically significant and associated with MDR. The identification of plasmid‐borne *bla*_*NDM*_ and *mcr* genes, in particular, raises concerns about the potential dissemination of resistance traits through the food chain. This finding highlights the need for routine surveillance to prevent ARG dissemination through foodborne pathogens, posing risks to public health and emphasizing the importance of stringent antimicrobial stewardship in livestock farming. Additionally, some ARGs in the bacteria detected in our study may not expressed, which could be due to environmental factors or the specific conditions under which these bacteria were isolated. While these unexpressed ARGs may pose a lower immediate risk, they could potentially be activated in different environments, such as the human gut, contributing to antimicrobial resistance. Further investigation into the expression of these genes is needed to assess their full impact on public health.

### Examination of abundance and diversity of VFGs and MGEs in foodborne pathogens on pork surfaces

Metagenomic analysis identified 13 types of VFGs across all samples, encompassing functions such as adherence, biofilm formation, immune modulation, and stress survival mechanisms, with subtypes ranging from 208 to 1017 (Tables S5, S6). PK1‐1, PK1‐2, and PK2‐1 exhibited over 606 VFG genes, whereas PK3‐1, PK3‐3, and PK3‐4 had significantly fewer. Six types of mobile genetic elements (MGEs), including integrative and conjugative elements, transposons, and insertion sequences, were detected, with PK1‐2, PK1‐3, and PK2‐1 containing over 1000 subtypes, while PK3‐3 had only 411. Dominant MGE genes included ISAs31, Tn6216, and ISAba8. Isolate‐based analysis revealed diverse VFG and MGE profiles across bacterial species, with *E. coli* showing the highest diversity (10 VFG types, 3841 subtypes). A total of 200 VFG genes, primarily from the effector delivery system, were uniquely identified in isolates. Species such as *K. aerogenes* and *S. enterica* harbored distinct genes like *allA*, *hilC*, and *pltB*. Unique MGEs, including Tn3411 in *Klebsiella spp*. and Tn558 in *E. faecalis* plasmids, were observed. Comparatively, isolate genomes revealed 55 MGE subtypes not found in metagenomic data, emphasizing their complementarity. The diversity of MGEs and VFGs highlights their role in bacterial adaptation and pathogenicity. Identifying unique genes, such as *hilC* in *S. enterica*, underscores the potential for horizontal gene transfer and persistence in foodborne bacteria. Future research will investigate the roles of VFGs and MGEs in bacterial adaptation and virulence, with an emphasis on their potential for horizontal gene transfer and persistence in foodborne pathogens.

### MAGs on the surface of pork

Thirteen MAGs were recovered from five samples: PK1‐1 (*n* = 5), PK1‐2 (*n* = 4), PK1‐3 (*n* = 1), PK1‐4 (*n* = 2), and PK3‐4 (*n* = 1) (Figure S1). Most MAGs exhibited over 70% completeness and less than 10% contamination, spanning three phyla and six genera: *Corynebacterium*, *Lactiplantibacillus*, *Weissella*, *Macrococcus*, *Staphylococcus*, and *Escherichia*. Two MAGs of *Corynebacterium variabile* were recovered from PK1‐1 and PK1‐2, carrying the *icl* VFG. *Lactiplantibacillus plantarum*, assembled from PK3‐4, is a common foodborne pathogen, while *Weissella cibaria*, detected in PK1‐1, is typically associated with food fermentation. Three MAGs of *Macrococcus caseolyticus*, identified from different samples at the same location, included one carrying the *tet(M)* antibiotic resistance gene. Similarly, three MAGs of *Staphylococcus kloosii* were identified, with one harboring the *blaZ* gene. Lastly, a single *E. coli* MAG from PK1‐4 contained 49 VFGs and exhibited notable genomic diversity. These MAGs reveal diverse microbial populations on pork surfaces, including pathogens and commensals. The 13 MAGs presented in this study, including *Corynebacterium variabile* and *Weissella cibaria*, may play a role in foodborne contamination. While these species are not commonly associated with foodborne illness, the presence of VFGs within their genomes, such as *icl*, suggests that they could contribute to pathogenicity in the food chain. In our future studies, we will focus on characterizing unclassified MAGs and their genomes to explore their potential roles in antimicrobial resistance, virulence, and overall contributions to the microbiome on pork surfaces.

Finally, wet markets are monitored through periodic inspections of hygiene, food handling, and pathogen testing. However, routine surveillance of ARGs, VFGs, and resistant pathogens is lacking. We recommend implementing molecular screening for ARGs and VFGs, enforcing stricter hygiene regulations, and enhancing consumer education to mitigate risks associated with wet market food.

## AUTHOR CONTRIBUTIONS

**Lianwei Ye**: Conceptualization; methodology; software; data curation; investigation; validation; writing—original draft; formal analysis; visualization. **Qiao Hu**: Investigation; validation. **Tao Zang**: Investigation. **Yaling Wang**: Investigation. **Heng Heng**: Data curation; software. **Edward Wai Chi Chan**: Writing—review and editing.

## CONFLICT OF INTEREST STATEMENT

The authors declare no conflicts of interest.

## ETHICS STATEMENT

No animals or humans were involved in this study.

## Supporting information


**Figure S1.** Phylogenetic tree of Isolation and MAGs.

**Table S1.** Collection sites and geographical coordinates of pork samples.
**Table S2.** Relative abundance of various bacterial phyla across different samples.
**Table S3.** Relative abundance of various bacterial genus across different pork samples (%).
**Table S4.** Antibiotic resistance profile of pork samples.
**Table S5.** Mobile element gene profile of pork samples.
**Table S6.** Virulence gene profile of pork samples.

## Data Availability

All the raw data used in this research are available in the NCBI Sequence Read Archive (SRA) database with BioProject of PRJNA1080313 at: https://www.ncbi.nlm.nih.gov/bioproject/PRJNA1080313. The data and scripts used are saved in GitHub (https://github.com/LianweiYe/pork-surface-microbiome). Supplementary materials (methods, figures, tables, graphical abstract, slides, videos, Chinese translated version, and update materials) may be found in the online DOI or iMetaOmics http://www.imeta.science/imetaomics/. The data that support the findings of this study are openly available in NCBI at https://www.ncbi.nlm.nih.gov/bioproject/?term=PRJNA1080313, reference number PRJNA1080313.
