# Supplementary material for: Deciphering comprehensive profiles of pathogenies and resistome of pork using integrating metagenomic and isolation strategies
Source: IMetaOmics. 2025 Feb 25;2(2):e70004. doi: 10.1002/imo2.70004 (PMC12806203; doi:10.1002/imo2.70004)
Supplement: Supplementary file 1 — Figure S1. Phylogenetic tree of Isolation and MAGs. [file IMO2-2-e70004-s002.docx]

**Supporting information to Deciphering comprehensive profiles of pathogenies and resistome of pork using integrating metagenomic and isolation strategies**

**Running title: Pathogen and Resistome Pork Profiling**

Lianwei YE^1,2^, Qiao Hu ^1,2^, Tao Zang^2,3^, Yaling Wang^3^, Heng HENG^1,2^, Edward Wai Chi CHAN^2^, Sheng CHEN^2,3*^

^1^Department of Infectious Diseases and Public Health, Jockey Club College of Veterinary Medicine and Life Sciences, City University of Hong Kong, Kowloon, Hong Kong 999077, China

^2^State Key Lab of Chemical Biology and Drug Discovery and the Department of Food Science and Nutrition, The Hong Kong Polytechnic University, Hung Hom, Kowloon, Hong Kong 999077, China

^3^Shenzhen Key Lab for Biological Safety Control, The Hong Kong Polytechnic University Shenzhen Research Institute, Shenzhen 518000, China

*Correspondence: sheng.chen@polyu.edu.hk (Sheng Chen)

**Methods**

**The isolation of HPB and conducting antimicrobial susceptibility tests**

From 10 pork samples collected from various locations in Hong Kong, including MongKok Market, Ho Hing Sun Kee Meat Company, and New Generation Fruits & Vegetables, a variety of bacterial species were isolated. The species identified include *A. pittii* (*n* = 2), *A. dhakensis* (*n* = 2), *A. veronii* (*n* = 2), *E. kobei* (*n* = 1), *E. faecalis* (*n* = 2), *E. gallinarum* (*n* = 1), *E. coli* (*n* = 51), *K. aerogenes* (*n* = 2), *K. pneumoniae* (*n* = 6), *M. morganii* (*n* = 3), *P. putida* (*n* = 1), *S. enterica* (*n* = 2), and *S. bockelmannii* (*n* = 1). The specific locations were identified by the following coordinates: PK1 (22.322269327019455, 114.17109463981373), PK2 (22.305940891611154, 114.16926251298473), and PK3 (22.304151756212267, 114.16894402119749). The samples underwent standard processing procedures, as previously described [1].

Adjusting the methodology, antibiotic plates were prepared with tigecycline (4 mg/L), and meropenem (0.5 mg/L). Different types of agar plates, such as Thiosulfate Citrate Bile Salts Sucrose agar, Baird-Parker agar, Xylose Lysine Tergitol-4 agar, and MacConkey agar, were utilized for isolation purposes. Suspected colonies were identified using Matrix-Assisted Laser Desorption/Ionization Time-of-Flight Mass Spectrometry (MALDI-TOF MS). The AST guidelines referenced are from the Clinical and Laboratory Standards Institute (CLSI) document M100, 32nd Edition, which provides detailed methods for antimicrobial susceptibility testing [2]. The isolates underwent MIC testing across 10 major classes of antibiotics, including Aminoglycosides, β-lactamase Inhibitors, Carbapenems, Cephalosporins, Chloramphenicol, Penicillins, Polymyxins, Quinolones, Sulfonamides, and Tetracyclines. Additionally, 14 subcategories were tested, comprising Ampicillin (AMP), Amikacin (AMK), Amoxicillin-Clavulanate (AMC), Cefotaxime (CTX), Cefoxitin (FOX), Ceftriaxone (CRO), Chloramphenicol (CHL), Ciprofloxacin (CIP), Colistin (CLS), Gentamicin (GEN), Meropenem (MRP), Nalidixic Acid (NAL), Sulfamethoxazole (SXT), and Tigecycline (TIG). For colistin, following international consensus, the microbroth dilution method was used for susceptibility testing, as it is the recommended standard for this drug, rather than the agar dilution method.

**Sequencing of the entire genome and subsequent bioinformatics analysis**

Genomic DNA extraction was conducted utilizing the PureLink Genomic DNA Minikit from Invitrogen (USA), following the manufacturers' instructions. Short-read sequencing of the genomic DNA was carried out on the Illumina Hiseq X platform for 300 cycles (250 bp paired-end). Data preprocessing and obtaining clean data involved several steps using SOAPnuke filtering parameters: "-n 0.01 -1 20 -g 0.4 --adaMis 3 --outQualSys 1 --minReadLen 150." The filtering steps included adapter trimming, read length filtering, N-base filtering, and low-quality data filtering. Illumina short reads underwent de novo assembly via SPAdes v4.0.0 [3], with discarding of contigs <500 bp. To identify and annotate resistance genes, mobile elements, and other unique genetic features, CARD, ResFinder [4], and ISfinder were employed. PLASMe v1.1 was utilized to identify plasmids[5]. To support the claims regarding the relative abundance of pathogens and ARGs across samples, we have included P-values for all relevant comparisons. Statistical analyses were performed using Kruskal-Wallis, with significance levels indicated for key findings in the text and figures. These results ensure a more robust interpretation of the data.

**Extraction and sequencing of metagenomic DNA**

The pork samples underwent aseptic dissection using sterile scissors. The dissected sections were finely minced and then transferred into either sterile sample bags or homogenization bags. Each homogenization bag was supplied with 50 mL of sterile 0.85% saline solution or PBS buffer, and the contents were homogenized until a paste-like consistency was achieved using a sterile homogenizer. The mixture was subjected to filtration using an 8μm filter to separate bacterial cells, and the resulting filtrate was collected as the supernatant. Subsequently, centrifugation at 8,000 × g for 8 minutes was performed to pellet the bacterial cells, yielding the desired bacterial pellet. The centrifuged suspension yielded a bacterial pellet, from which a segment (200 μL) was diluted in PBS for bacterial isolation. Simultaneously, another segment (180 μL) was allocated for metagenomic DNA extraction. This method ensures that both metagenomic analysis and bacterial isolation are conducted on samples derived from identical portions of the pork, enabling a direct comparison of the resultsDNA extraction from the bacterial pellet was conducted using the QIAamp PowerFecal Pro DNA Kit as per the manufacturer's instructions. Subsequent genomic DNA extraction utilized the QIAGEN Genomic-tip 100/G following the manufacturer’s guidelines. Assessment of DNA concentration and purity was conducted using the Qubit DNA Assay Kit with a Qubit 2.0 Fluorometer for concentration and a Nanodrop 2000 for purity evaluation. Genomic DNA underwent fragmentation through Frag enzyme treatment, yielding 350bp-sized fragments. These fragments underwent successive processes including end-polishing, A-tailing, and adaptor ligation to enable DNB sequencing, followed by PCR amplification. PCR products were denatured and ligated with a complementary molecule using DNA ligase. The resulting linear molecules were digested with exonuclease to produce single-strand circular DNA libraries. Library quality was analyzed using Qubit for quantification, real-time PCR, and a bioanalyzer for size distribution assessment. Quantified libraries were evenly combined to form DNA Nanoballs (DNB) and sequenced on a DNBseq-T7 platform, generating 20 G of raw data per sample with a PE150 read length.

**Assembly of metagenomes, prediction of genes, taxonomic assignments, and statistical analysis**

For initial preprocessing and quality control of the raw sequences, Trimmomatic (version 0.39) was employed. This process included removing adapter sequences, discarding reads shorter than 36 bases, and filtering out reads with quality scores below 15. To eradicate host contamination from the shotgun metagenomic data, KneadData (v0.7.7) was utilized. After preprocessing and quality control, the metagenomic reads were aligned against marker genes from microbial reference genomes using Bowtie2 (version 2.5.1) via the MetaPhlAn4 package. Subsequently, assembly of the cleaned reads was performed using megahit v1.2.9 with default parameters [6-8]. Taxonomic classification and abundance estimation were carried out using MetaPhlan4. For functional gene analysis, including resistome profiling, we used ARGs-OAP v2.2 with a sequence identity threshold of ≥ 90% and a minimum alignment length of ≥ 80%. These parameters were selected based on published recommendations to ensure high specificity and reduce false positives. Identified ARGs were further validated by cross-referencing with curated databases such as CARD and ResFinder. This multi-step approach minimized potential errors and provided confidence in the identification of antimicrobial resistance genes.

Moreover, analysis of MEG, and VFG diversity and abundance was conducted using the ARGs-OAP pipeline, leveraging the VFGs, and MEGs databases, respectively [9, 10]. Species identification was performed by cross-referencing two databases to identify HPB at the species level. These included an established pathogen database[11] and the official lists of pathogenic microorganisms transmissible among humans in China, as published by the National Health Commission (NHC) of the People's Republic of China (<http://www.nhc.gov.cn/qjjys/s7948/202308/b6b51d792d394fbea175e4c8094dc87e.shtml>)

**Figure S1**
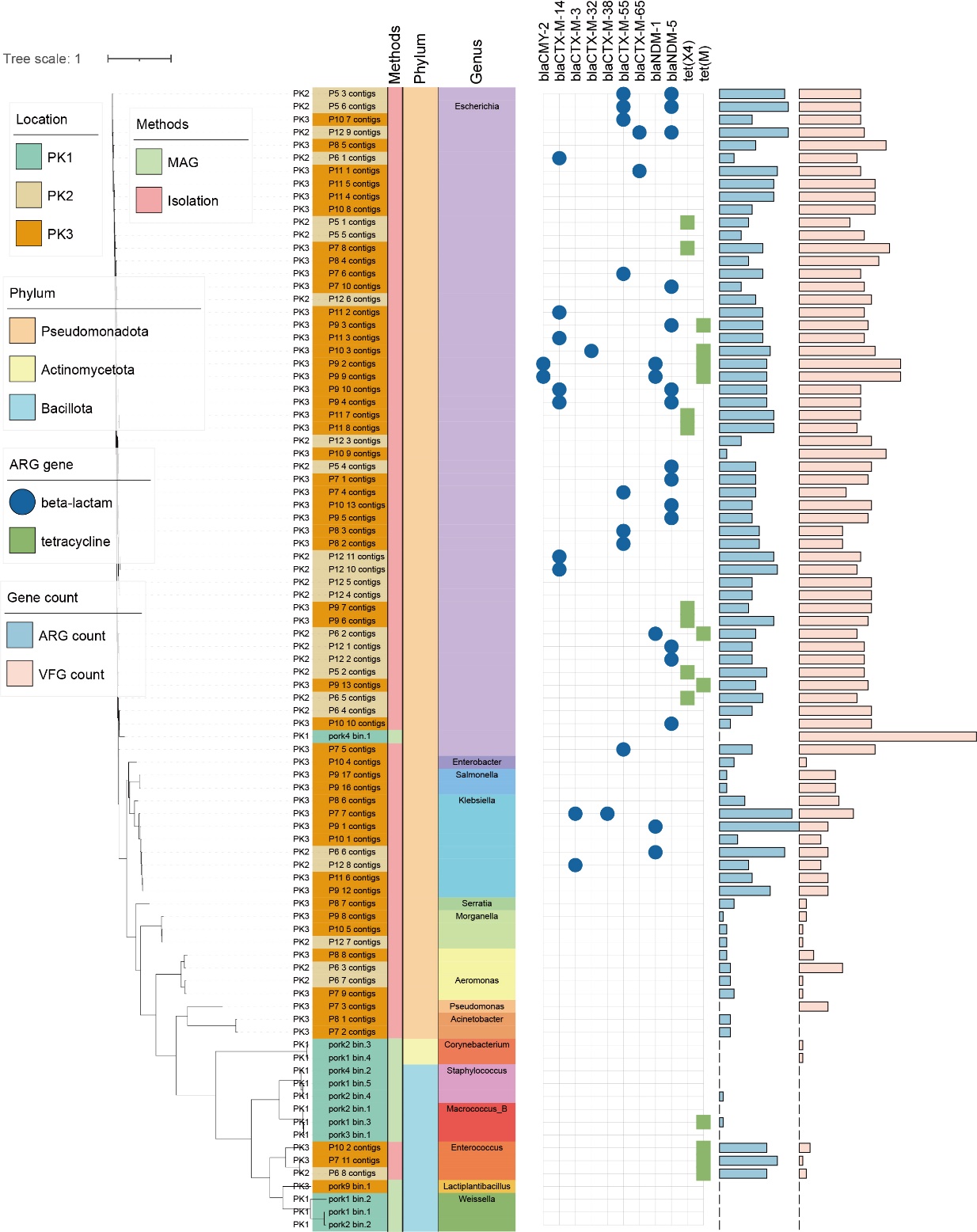
 **Phylogenetic tree of Isolation and MAGs**. This figure illustrates the distribution of ARG genes across different phyla, identified using various location methods. The X-Axis represents the gene count, including both ARG and VFG counts. The heatmap colors indicate specific phyla, while the shapes represent distinct ARG genes identified at each location. The labels on the left include “Location” with PK1, PK2, and PK3; “Methods” with MAG and Isolation; “*Phylum*” with Pseudomonadota, Actinomycetota, and Bacillota; and “ARG gene” with beta-lactam and tetracycline. To the right of the heatmap are two bar graphs representing “ARG count” and “VFG count” respectively for each row in the heatmap. Each row in both sections (heatmap and bar graphs) corresponds to a specific combination of location method, phylum type, and ARG gene. The tree scale at the bottom left corner indicates hierarchical clustering or phylogenetic relationships among different samples or species.

**REFERENCES**

1. Li, Ruichao, Lianwei Ye, Marcus Ho Yin Wong, Zhiwei Zheng, Edward Wai Chi Chan, Sheng Chen. 2017. “Evolution and comparative genomics of pAQU-like conjugative plasmids in Vibrio species.” *Journal of Antimicrobial Chemotherapy* 72: 2503-2506. <https://doi.org/https://doi.org/10.1093/jac/dkx193>

2. Jorgensen, James H, Janet F Hindler, L Barth Reller, Melvin P Weinstein. 2007. “New consensus guidelines from the Clinical and Laboratory Standards Institute for antimicrobial susceptibility testing of infrequently isolated or fastidious bacteria.” *Clinical infectious diseases* 44: 280-286. <https://doi.org/https://doi.org/10.1086/510431>

3. Bankevich, Anton, Sergey Nurk, Dmitry Antipov, Alexey A Gurevich, Mikhail Dvorkin, Alexander S Kulikov, Valery M Lesin, Sergey I Nikolenko, Son Pham, Andrey D Prjibelski. 2012. “SPAdes: a new genome assembly algorithm and its applications to single-cell sequencing.” *Journal of computational biology* 19: 455-477. <https://doi.org/https://doi.org/10.1089/cmb.2012.0021>

4. Bortolaia, Valeria, Rolf S Kaas, Etienne Ruppe, Marilyn C Roberts, Stefan Schwarz, Vincent Cattoir, Alain Philippon, Rosa L Allesoe, Ana Rita Rebelo, Alfred Ferrer Florensa. 2020. “ResFinder 4.0 for predictions of phenotypes from genotypes.” *Journal of Antimicrobial Chemotherapy* 75: 3491-3500. <https://doi.org/https://doi.org/10.1093/jac/dkaa345>

5. Tang, Xubo, Jiayu Shang, Yongxin Ji, Yanni Sun. 2023. “PLASMe: a tool to identify PLASMid contigs from short-read assemblies using transformer.” *Nucleic acids research* gkad578. <https://doi.org/https://doi.org/10.1093/nar/gkad578>

6. Koren, Sergey, Brian P Walenz, Konstantin Berlin, Jason R Miller, Nicholas H Bergman, Adam M Phillippy. 2017. “Canu: scalable and accurate long-read assembly via adaptive k-mer weighting and repeat separation.” *Genome research* 27: 722-736. <https://doi.org/10.1101/gr.215087.116>

7. Nurk, Sergey, Dmitry Meleshko, Anton Korobeynikov, Pavel A Pevzner. 2017. “metaSPAdes: a new versatile metagenomic assembler.” *Genome research* 27: 824-834. <https://doi.org/10.1101/gr.213959.116>

8. Li, Dinghua, Chi-Man Liu, Ruibang Luo, Kunihiko Sadakane, Tak-Wah Lam. 2015. “MEGAHIT: an ultra-fast single-node solution for large and complex metagenomics assembly via succinct de Bruijn graph.” *Bioinformatics* 31: 1674-1676. <https://doi.org/https://doi.org/10.1093/bioinformatics/btv033>

9. Yang, Ying, Xiaotao Jiang, Benli Chai, Liping Ma, Bing Li, Anni Zhang, James R Cole, James M Tiedje, Tong Zhang. 2016. “ARGs-OAP: online analysis pipeline for antibiotic resistance genes detection from metagenomic data using an integrated structured ARG-database.” *Bioinformatics* 32: 2346-2351. <https://doi.org/https://doi.org/10.1093/bioinformatics/btw136>

10. Liu, Bo, Dandan Zheng, Siyu Zhou, Lihong Chen, Jian Yang. 2022. “VFDB 2022: a general classification scheme for bacterial virulence factors.” *Nucleic acids research* 50: D912-D917. <https://doi.org/https://doi.org/10.1093/nar/gkab1107>

11. Li, Bing, Feng Ju, Lin Cai, Tong Zhang. 2015. “Profile and fate of bacterial pathogens in sewage treatment plants revealed by high-throughput metagenomic approach.” *Environmental science & technology* 49: 10492-10502. <https://doi.org/https://pubs.acs.org/doi/abs/10.1021/acs.est.5b02345>
